# A Proposed Human Structural Brain Connectivity Matrix in the Center for Morphometric Analysis Harvard-Oxford Atlas Framework: A Historical Perspective and Future Direction for Enhancing the Precision of Human Structural Connectivity with a Novel Neuroanatomical Typology

**DOI:** 10.1159/000530358

**Published:** 2023-03-28

**Authors:** Nikos Makris, Richard Rushmore, Jonathan Kaiser, Matthew Albaugh, Marek Kubicki, Yogesh Rathi, Fan Zhang, Lauren J. O’Donnell, Edward Yeterian, Verne S. Caviness, David N. Kennedy

**Affiliations:** a Center for Morphometric Analysis, Harvard Medical School, Massachusetts General Hospital, Boston, MA, USA; b Psychiatry Neuroimaging Laboratory, Harvard Medical School, Brigham and Women’s Hospital, Boston, MA, USA; c Department of Anatomy and Neurobiology, Boston University School of Medicine, Boston, MA, USA; d Department of Psychiatry, University of Vermont Larner College of Medicine, Burlington, VT, USA; e Department of Radiology, Brigham and Women’s Hospital and Harvard Medical School, Boston, MA, USA; f Department of Psychology, Colby College, Waterville, ME, USA; g Department of Psychiatry, University of Massachusetts Chan Medical School, Worcester, MA, USA

**Keywords:** Structural brain connectivity matrix, Human, Harvard-Oxford Atlas, MRI, Diffusion tensor imaging

## Abstract

A complete structural definition of the human nervous system must include delineation of its wiring diagram (e.g., Swanson LW. Brain architecture: understanding the basic plan, 2012). The complete formulation of the human brain circuit diagram (BCD [Front Neuroanat. 2020;14:18]) has been hampered by an inability to determine connections in their entirety (i.e., not only pathway stems but also origins and terminations). From a structural point of view, a neuroanatomic formulation of the BCD should include the origins and terminations of each fiber tract as well as the topographic course of the fiber tract in three dimensions. Classic neuroanatomical studies have provided trajectory information for pathway stems and their speculative origins and terminations [Dejerine J and Dejerine-Klumpke A. Anatomie des Centres Nerveux, 1901; Dejerine J and Dejerine-Klumpke A. Anatomie des Centres Nerveux: Méthodes générales d’étude-embryologie-histogénèse et histologie. Anatomie du cerveau, 1895; Ludwig E and Klingler J. Atlas cerebri humani, 1956; Makris N. Delineation of human association fiber pathways using histologic and magnetic resonance methodologies; 1999; Neuroimage. 1999 Jan;9(1):18–45]. We have summarized these studies previously Neuroimage. 1999 Jan;9(1):18–45] and present them here in a macroscale-level human cerebral structural connectivity matrix. A matrix in the present context is an organizational construct that embodies anatomical knowledge about cortical areas and their connections. This is represented in relation to parcellation units according to the Harvard-Oxford Atlas neuroanatomical framework established by the Center for Morphometric Analysis at Massachusetts General Hospital in the early 2000s, which is based on the MRI volumetrics paradigm of Dr. Verne Caviness and colleagues [Brain Dev. 1999 Jul;21(5):289–95]. This is a classic connectional matrix based mainly on data predating the advent of DTI tractography, which we refer to as the “pre-DTI era” human structural connectivity matrix. In addition, we present representative examples that incorporate validated structural connectivity information from nonhuman primates and more recent information on human structural connectivity emerging from DTI tractography studies. We refer to this as the “DTI era” human structural connectivity matrix. This newer matrix represents a work in progress and is necessarily incomplete due to the lack of validated human connectivity findings on origins and terminations as well as pathway stems. Importantly, we use a neuroanatomical typology to characterize different types of connections in the human brain, which is critical for organizing the matrices and the prospective database. Although substantial in detail, the present matrices may be assumed to be only partially complete because the sources of data relating to human fiber system organization are limited largely to inferences from gross dissections of anatomic specimens or extrapolations of pathway tracing information from nonhuman primate experiments [Front Neuroanat. 2020;14:18, Front Neuroanat. 2022;16:1035420, and Brain Imaging Behav. 2021;15(3):1589–1621]. These matrices, which embody a systematic description of cerebral connectivity, can be used in cognitive and clinical studies in neuroscience and, importantly, to guide research efforts for further elucidating, validating, and completing the human BCD.

## Introduction

A complete structural definition of the human nervous system must include delineation of its wiring diagram (e.g., [1]). A comprehensive neuroanatomic formulation of the brain circuit diagram (BCD [2]) should elucidate how each part works in isolation and how its different components work in an integrative manner. In the words of the late Dr. Verne Caviness from the early 2000s:

“Herein we are taking into serious consideration the paradigm framework of the connectionist model. Nevertheless, it goes far beyond what routine clinical and pathological analysis does. This is because its analyses are both strictly quantitative in nature and also topographical in terms of relative location between brain structures and lesions as well as with respect to coordinate systems of reference. Traditional clinical-pathological correlations examine lesions in qualitative terms such as “this has knocked-out this area and that has knockedout that area” but none of these has looked quantitatively at how much brain and with respect to the specific structures of the brain in terms of “how much” has been involved in a neuropathological process. This keeps central the objective of testing the connectionist model and to understand its limitations. This is because knowledge of brain connections is indispensable to understanding function. However, knowledge of connections does not necessarily allow an understanding about that function. What we really need is an understanding to set-up circuitry, the way in which circuits are organized and even beyond that is the way in which they function physiologically.”

This reflection by Dr. Caviness can be interpreted as an effort to elucidate one of the most difficult aspects of human neuroanatomy, namely, structural connectivity (e.g., [3]). The first attempt to reveal a comprehensive blueprint of human structural brain connectivity using MRI was done at the Center for Morphometric Analysis of Massachusetts General Hospital and reported in seminal papers in the domain of MRI-based volumetrics [4-8]. Although incomplete, and perhaps naïve in appearance, this work reflected the thinking of traditional neuroanatomy of the past two centuries just before the availability of diffusion tensor imaging (DTI) tractography (e.g., [9-12]). We refer to this canon of knowledge as the “pre-DTI era” human structural connectivity matrix, or simply the pre-DTI era. With the advent of DTI tractography [13-15], the field of structural connectivity has grown exponentially in what we term the “DTI era” of the human structural connectivity matrix, or simply the DTI era. Dr. Caviness had a strong neuroanatomical foundation in the pre-DTI literature but also was present at the introduction of DTI techniques that revolutionized human brain structural connectivity studies [4, 5, 14].

### Overview of Structural Connectivity

In the mature cerebrum, connections are mediated via specific fiber pathways and are precisely organized in relation to specific architectonic areas. Brain architecture can be described in terms of different scales. A “microscale” (microscopic or ultrastructural level) description at the level of the synapse is essential for elucidating the underpinnings of somal and axodendritic architecture (e.g., [16]). Another level of explanation for neuroanatomical organization is that of regional cytoarchitectonics and connections. We refer herein to this level as “macroscale,” which describes brain regions or areas, e.g., Brodmann areas, and their associated fiber pathways. The macroscale level is highly useful for correlating anatomy with specific behaviors and functions and can be addressed with current in vivo neuroimaging techniques (e.g., [16, 17]). Based on literature mining, curation, and databasing focused on invasive tract tracing methodologies, a macroscale level of cerebral connectivity has been elucidated comprehensively in nonhuman vertebrates such as the macaque (e.g., [18-21]), rat, and mouse (e.g., [1]).

### Pre-DTI Era Human Structural Connectivity Data

To date, a set of white matter pathways in humans has been delineated with confidence using traditional histological techniques (e.g., [10]). These are the fiber pathways dedicated to motor and sensory functions, which have immediate clinical relevance and are a fundamental component of the routine neurological examination in a clinical setting. From a quantitative perspective, however, these pathways constitute only about 10% of the total cerebral white matter. The rest is made up of corticocortical ipsilateral associational fiber pathways and commissural bundles [4, 8]. Thus, 90% of fibers in the cerebrum deal with cognitive and emotional functioning and have been a relatively neglected part of brain structure in clinical neurology. The precise origins and terminations of these fibers are not determined in humans; only their stems have been delineated in postmortem material and more recently with DTI tractography. These fiber tracts in humans have been identified and traced in postmortem material using techniques such as microdissection of white matter (e.g., the Klingler technique [11, 22]), and the Waller [23, 24], Weigert [25, 26], Dejerine and Dejerine-Klumpke [9, 10], Marchi [27, 28], and Nauta silver staining techniques [29, 30]. Such methods have allowed for only a limited understanding of human brain structural connectivity and do not show the precise origins and terminations of fiber pathways (see [2] for review). There are currently available approaches in humans that allow the delineation of the origin, stem, or trajectory, and termination of a brain pathway, but these are limited to relatively short distances, typically 5 mm–1.5 cm [31-35]. Other methods in use include 3D polarized light imaging [36-40] as well as optical coherence tomography [41, 42] and expansion microscopy [43, 44]. Although promising, these techniques in humans do not enable the complete delineation of brain pathways as do experimental, invasive tract tracing methods in nonhuman primates [2].

### DTI Era Human Structural Connectivity Data and Its Challenges

DTI tractography is currently a major source of information in our understanding of human anatomic connectivity, making it a valuable instrument for comparative or validation studies of structural connectivity in humans [2]. That said and as elaborated upon above, knowledge of human structural cerebral connections in their entirety remains sparse and is hindered by methodological obstacles. DTI has enabled investigations of human brain connections; however, to date, DTI is too coarse to elucidate completely the macroscale level of cerebral connectivity, particularly in terms of precise areas of origin and termination (e.g., [2]). Specifically, using DTI-based segmentation and DTI tractography, numerous groups have been able to delineate the stems, i.e., the distinct white matter fascicles [14], of the principal fiber pathways in the brains of experimental animals (e.g., [45]) and humans (e.g., [15, 46-49]). DTI-based fiber tracking techniques, however, have not yet demonstrated the ability to define precisely the origins and terminations of specific fiber pathways [2]. Thus, the generation of a comprehensive set of fiber tracts in the experimental animal or human brain has not yet been achieved using DTI. Clearly, DTI tractography has revolutionized the field of human structural connectivity as reflected in the large number of publications during the past two decades. Nevertheless, these data have seldom been validated, as has been elaborated upon by Rushmore et al. [2]. The issue of validation has been addressed by other investigators as well, such as Deepak Pandya [45, 50] and Marsel Mesulam [51].

Given the lack of complete pathway information in the human brain, a comparison of human cerebral connections with homologous fiber pathways derived from hodological experiments in monkeys is a necessary step for determining a first approximation of the origins and terminations of fiber tracts (e.g., [2, 4, 45]). However, this is not a sufficient condition given the significant behavioral, structural, and allometric differences that exist between the two species and also bearing in mind that the cerebral cortex is the brain structure most affected by evolutionary processes (e.g., [4, 52-56]). Among the textbooks, monographs, and atlases that portray the array of fiber tracts in human brains (e.g., [9-11, 57]), Dejerine and Dejerine-Klumpke [9, 10] presented the most complete work on human fiber tracts. The Dejerines’ inferences from fixation techniques and lesion analysis in postmortem studies allowed them to delineate the stems of the principal fiber tracts in the human cerebrum at a gross level. Importantly, their observations included association fiber pathways. The work of Ludwig and Klingler [11] using fiber microdissection also revealed the stems of major pathways in the human brain. This technique has been further improved, especially in the hands of neurosurgeons (e.g., [58, 59]), and has allowed us to enrich our understanding of cortical white matter pathway stems. Nevertheless, in light of currently available knowledge, a study of human brain connections should make use of experimental, nonhuman primate material as a source of inferences regarding the origin and termination of fiber tracts. At the same time, it should take into account the relatively limited available human findings.

We have previously formulated a comprehensive quantitative MRI-based parcellation system for the white matter of the human brain, which subdivides the cerebral white matter into 49 superficial and 19 deep white matter parcellation units (PUs) per hemisphere [4, 8]. In addition, we have created maps of cerebral connectivity of the human cerebrum associated with these PUs [4]. Mapping of the fiber systems was based on both nonhuman experimental material and human connectional information derived from textbooks and atlases available for humans. This systematic mapping was part of the Center for Morphometric Analysis neuroanatomical volumetric framework (e.g., [4-8, 46, 60-63], which generated the original Harvard-Oxford Atlas (HOA [6, 60, 64]). It should be noted that the present manuscript refers to that original version of the HOA. In terms of the predictive value of this structural connectional framework for functional mapping, it is an ongoing assumption that this system will be continuously assessed and refined using different sources of information such as inferences from anatomical-clinical correlations and multimodal imaging [4, 5]. This framework considers the anatomic individuality of the brain under investigation as well as the topography of the different white matter fiber pathways within specific, volumetrically quantifiable PUs. A shortcoming of this original framework is that it did not provide explicit systematic lists for each cortical region or PU of the putative cortical and subcortical areas sharing connections with a given PU.

In the present study, we provide a macroscale level of inferred human structural cerebral connectivity for the pre-DTI era as a function of PUs, in the form of matrices. A matrix in the present context is an organizational construct that embodies anatomical knowledge about cortical areas and connections. A connectivity matrix can be expressed in terms of origins and terminations (qualitative matrix) and can include topographical and quantitative information (topographic matrix and quantitative matrix). Furthermore, we present a new approach for DTI era connectional data. This is a conservative approach grounded in homology between humans and nonhuman primates and intended to minimize false positive and false negative connectional results using DTI tractography [2, 65]. We use a neuroanatomical typology to characterize different types of connections in the human brain, which provides a critical dimension for organizing the matrices and the prospective database. Although substantial in detail, the present matrices necessarily represent a partial collation of human structural connectivity findings. This is inevitable given that the sources of data relating to human structural connectional organization are limited largely to inferences from gross dissections of anatomic specimens or extrapolations of pathway tracing information from non-human primate experiments [2]. That said, these matrices embody a systematic description of cerebral connectivity that can be used in cognitive and clinical studies in neuroscience and, importantly, to enhance research efforts for further elucidating, validating, and completing the human BCD [2].

## Methods

The objectives of the present work were threefold: (1) to create a matrix structure containing direct and indirect inferences about human structural cerebral connectivity; (2) to establish the content of this matrix through the mining and curation of published data; and (3) to provide an exemplary sample application demonstrating the utility of this matrix, and the design of an internet brain connectivity database. Each of these objectives is presented in the following sections. The longer term objective is to create a more comprehensive matrix and a database that can be regularly updated and interrogated using a web interface.

### Matrix and Proposed Database Design

**Fig. 1.**
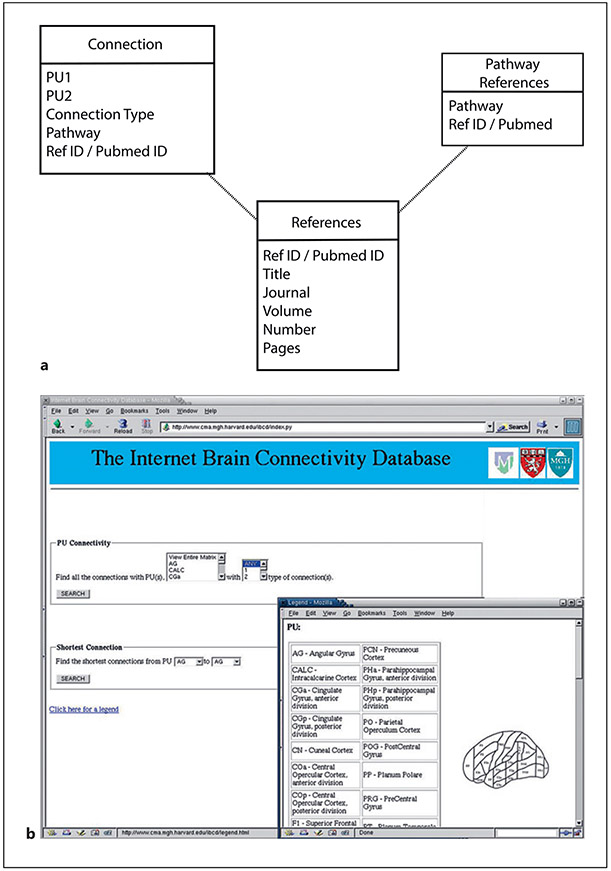
Proposed design of database schema and website. **a** The schema for the underlying database is provided. This simple infrastructure will provide database support for the literature-based observations of specific connections and pathways. **b** Front page of the proposed website. From this interface, queries by anatomic structure and by white matter pathway can be initiated.

In order to codify information from the literature on cerebral connectivity, we need a standardized expression for connectional information. The general form of expression we have chosen to use is “Region A is connected to Region B by Pathway C.” Each such assertion is supported by evidence in the form of one or more specific publications (and associated page, table, figure, tracer type, species, and other relevant information). In the classical pre-DTI era matrix, we include only published observations in human material in the manner reported by Dejerine and Dejerine-Klumpke (e.g., [4]). Each neuroanatomic region is associated with information such as the name of a specific cortical or subcortical area, associated Brodmann area(s), and associated functional systems, as reported by Rademacher et al. [60], Caviness et al. [6], and Makris et al. [4]. Although these are limited sources for such information, they serve as exemplars of supporting evidence for connectivity in the human brain. The goal of a complete and comprehensive connectional database would be to provide for each pathway a full range of information associated with it, including its preferred name and synonym(s) if any, its “class” of connection (local, associational, commissural, projection), and distance type (intragyral, juxtagyral, intralobar, long-range). The design of the database capturing this kind of information is summarized in Figure 1.

### Neuroanatomic Framework

In order to codify information from the literature on anatomic targets of connectivity, we need a neuroanatomic framework for the human brain. Thus, we have selected such a framework based on anatomy that can be precisely observed using in vivo imaging of humans, namely, the “cortical parcellation” framework introduced at the Center for Morphometric Analysis of Massachusetts General Hospital by Rademacher et al. [60] and operationalized and validated by Caviness et al. [5, 6]. This system has been used in numerous clinical and basic neuroscience studies investigating volumetric (e.g., [6, 66-73]) as well as cortical thickness and surface measurements (e.g., [74-77]). Furthermore, it has been implemented in functional MRI (e.g., [78, 79]), lesion analysis (e.g., [80-82]), and diffusion imaging (e.g., [4, 68, 71, 76, 83, 84]). Its implementations are both user-guided (e.g., [4, 6, 63, 85, 86]) and automated (e.g., [87, 88]). Moreover, this system has been used as the basis for a number of subsequent neuroanatomical variations (e.g., [86, 89-92]; see [92] for review). Inferences from the monkey literature can be made relative to putative homologies between monkey and human cortical and subcortical areas (e.g., [65, 93, 94]). Table 1 lists the complete set of human anatomic regions on which we have based this system. It should be noted that in the present framework and in the context of the typology detailed below, the insula is regarded as a distinct lobe as was described in the original HOA conceptualization by Rademacher, Caviness, and colleagues [60].

### Connection Class and Distance Type

**Fig. 2.**
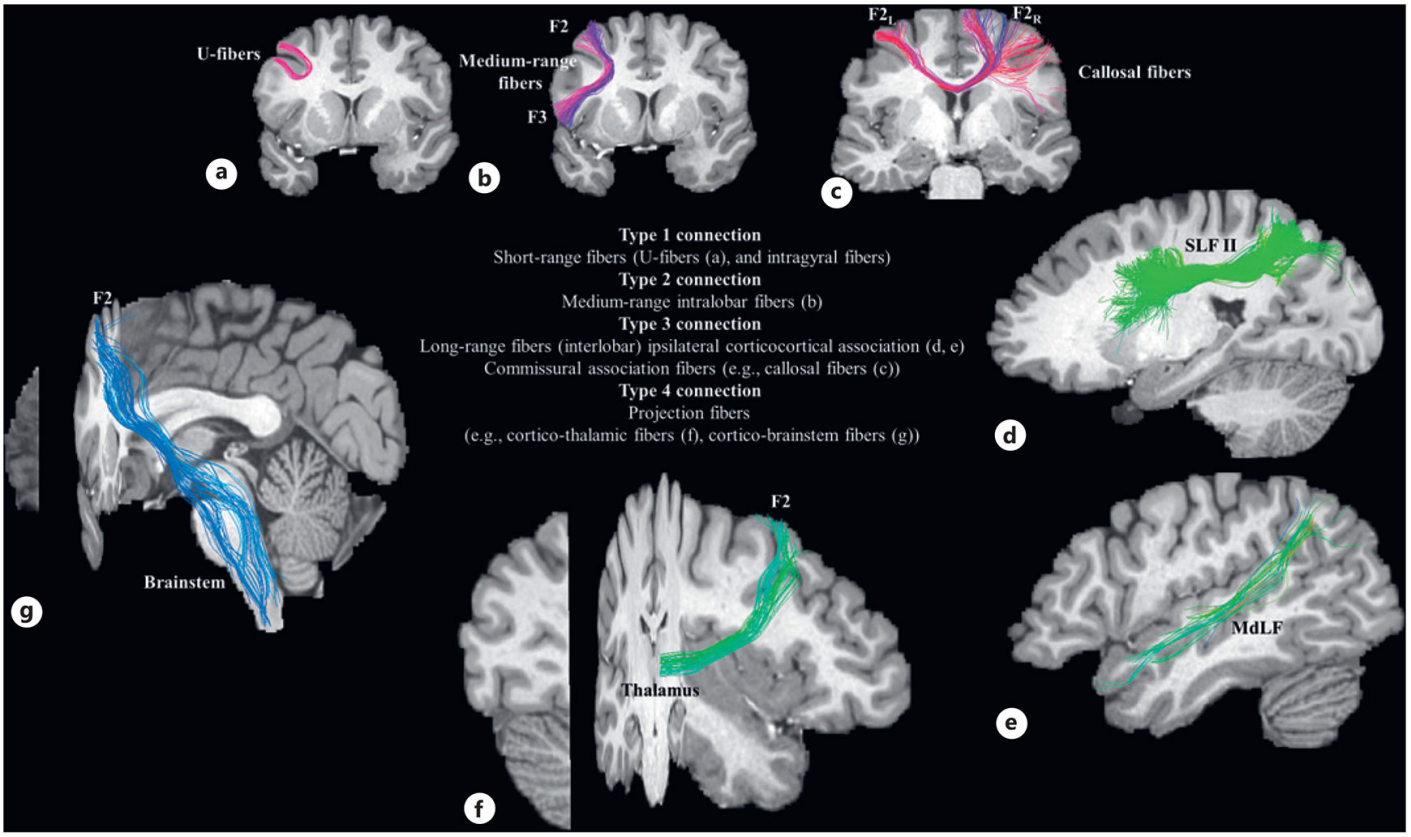
**a–g** This figure illustrates examples of the four types of distance connections (types 1,2, 3, 4) reconstructed using diffusion tensor imaging (DTI) tractography in a representative MRI dataset. F2, middle frontal gyrus; F2_L_, left middle frontal gyrus; F2_R_, right middle frontal gyrus; F3, inferior frontal gyrus; MdLF, middle longitudinal fascicle [68, 84, 179, 180]; SLF II, superior longitudinal fascicle II [46].

The white matter of the cerebral hemispheres that comprises the connectional matrix is the overall set of myelinated and unmyelinated axons, broadly conceived of as ipsilateral association, commissural, and projection fibers [14]. A key factor for the creation of a connectional matrix is the characterization of brain fiber pathways according to a neuroanatomical typology that reflects their nature. Association connections can also be described in terms of distance. Short-range fibers (type 1) connect adjacent areas. These fibers can connect areas within a lobe (intralobar) or adjacent cortical areas from two different lobes (juxtalobar). Medium-range fibers (type 2) refer to connections between non-adjacent areas within a lobe. Long-range (type 3) fibers connect cortical areas from two different lobes. These type 3 association connections include ipsilateral pathways within a hemisphere and also interhemispheric commissural connections. Finally, projection fibers that connect the cerebral cortex with subcortical structures are referred to as type 4. These various fiber types are listed in Table 2 and shown in Figure 2. According to the present definition and convention, type 1 and type 2 fibers for a PU cannot coexist with type 3 fibers. For example, the frontal pole PU cannot connect with its adjacent or intralobar PUs (e.g., superior frontal [F1], precentral gyrus, respectively) via type 3 fibers given that these PUs are within the frontal lobe and thus, by definition, interconnected by type 1 and 2 fibers.

Brain fiber pathways are distinctive in terms of their origins and terminations as well as the topography, trajectory, and size of their stems [4, 14]. Association fibers originate preferentially from cortical layers II and III of cerebral gyri and typically follow a curvilinear trajectory to terminate in layers II, III, and IV of target gyri. If the interconnected gyri are adjacent, such as the superior frontal gyrus (F1) and the middle frontal gyrus (F2), we assign the distance type 1 to denote “intragyral”/“juxtagyral” connections. If the specific brain areas are not adjacent but are in the same lobe, such as F1 and inferior frontal gyrus (F3), we assign the distance type 2 to denote an “intralobar” connection. By contrast, if the connected gyri are even more distant and are parts of different lobes, we assign the distance type 3 to denote a long-range “interlobar” connection. If known, we can also specify the particular long-range association fiber tract involved in that connection. This schema parallels the anatomic observation that the shortest and most superficial white matter fibers, or U-fibers, course beneath the fundus of a sulcus and curve around it in a U-shaped manner to terminate on the walls of two adjacent gyri (e.g., [10]). Thus, U-fibers are assigned the type juxtagyral (for short-range type 1) in this system. In addition, other fibers connect gyri located in more distant regions within the same lobe or different lobes of the hemisphere. These fibers, which course more deeply within the cerebral white matter and form compact stems of variable length, are assigned the type intralobar for medium-range (type 2) and “interlobar” for long-range connections (type 3).

Once codified, the complete set of connectional observations can be expressed in a matrix formulation, whereby the complete set of connections between each pair of anatomic targets can be indicated. An exemplar of this formulation is the *cortical connection matrix*, C, where:

$$C=\left[\begin{array}{ccccc} C_{1,1} & C_{1,2} & C_{1,3} & \ldots & C_{1,49}\\ C_{2,1} & C_{2,2} & C_{2,3} & \ldots & C_{2,49}\\ C_{3,1} & C_{3,2} & C_{3,3} & \ldots & C_{3,49}\\ C_{49,1} & C_{49,2} & C_{49,3} & \ldots & C_{49,49} \end{array}\right]$$

Each element, $C_{i,j}$ represents the structural connectivity information between anatomic regions i and j (note that there are 49 cortical regions in the localization system implemented and discussed here). These elements can be expressed as a 1 × 4 logical column vector indicating the presence (1) or absence (0) of connections, as well as pointers to the details of the connectional evidence, of the four distance types. The vertical order of the number in the vector indicates, from top to bottom, the presence or absence of connection types 1 through 4. For llexample, the prospective vector shown here

$$C_{F2,\text{AG}}=\left[\begin{array}{c} 0\\ 0\\ 3\\ 0 \end{array}\right]$$

indicates the absence of short-range intragyral/juxtagyral (type 1), medium-range intralobar (type 2), and projection (type 4) connections as well as the presence of long-range association (type 3) connections between the middle frontal gyrus (F2) and the angular gyrus (AG). The specific data in the matrix for this long-range association connection are drawn from anatomical sources and include details about the connection type (type 3) and anatomic name (e.g., superior longitudinal fascicle, SLF) (e.g., [46]).

It should be noted that the typology of human brain connections is identical in both the pre-DTI and DTI era matrices given that this typology reflects an organizational dimension of brain neuroanatomical connectivity. Given the paucity of data for type 1 and type 2 connections in experimental nonhuman primate studies, these connections in the human are not validated and thus have not been included in the present DTI era structural connectivity matrix. Type 4 connections, which are not considered in the present DTI era structural connectivity matrix, have been included in other sources (e.g., white matter query language software [95, 96]) for a limited number of projection pathways.

### Data Entry

The entry of connectional data is the most time-consuming aspect of database creation. There is a vast literature of observations on cerebral connections that precede the DTI era. This literature addresses ipsilateral cortico-cortical [10, 11, 14, 57, 97-114], commissural (e.g., callosal) (e.g., [45, 115-119]), corticothalamic (e.g., [120-122]), corticostriatal (e.g., [123, 124, 125, 126, 127, 128, 129, 130, 131, 132]), cortico-amygdaloid (e.g., [133, 134, 135, 136]), and cortico-hippocampal connectivity (e.g., [137, 138, 139, 140, 141, 142, 143, 144, 145]), as well as corticopontine pathways (e.g., [146, 147, 148, 149, 150, 151, 152, 153, 154]). For the cortico-cortical fiber tracts in particular, connectional relationships are inferred principally from the topographic characteristics of the fiber systems with regions of interest as collated by traditional pre-DTI neuroanatomists (e.g., [10, 11, 57, 108]). In the present study, these classic observations have been used as the basis for populating the pre-DTI matrix. Subsequently, Deepak Pandya (see [97]) observed that the general topography of the stems of major fiber tracts in the human brain has similarities to the stems of the same fiber pathways in the monkey brain and eventually translated this information to the field of neuroimaging [4]. This translation is especially important given that cortico-cortical fiber tracts in humans are not validated completely, as recently emphasized by our group [2]. More specifically, although close interspecies correspondences have been shown for the stem components of the fiber bundles, similarly rigorous comparisons regarding their origins and terminations in monkey and human brains remain to be established [2]. This unresolved aspect of human structural brain connectivity results in continuing uncertainty with respect to false positive and false negative findings arising from DTI tractography [2, 51]. Thus, the matrix proposed herein is based on pre-DTI era connectional information in the human brain derived from classical human neuroanatomy studies.

Our initial efforts in this area [4] were summarized in terms of the principal connections of the cerebrum and the anatomic nomenclature used therein. At the time, this effort covered classical and contemporary literature on human structural cerebral connectivity in over 100 original reports and monographs [4, 97] and we refer to these results as pre-DTI era data. The pre-DTI era matrix codifies evidence for specific pathways and their targets in the human brain. For the purpose of contrasting and showing differences between pre-DTI and DTI era connectional information in humans, we have included findings on cortical gray matter connectivity of two exemplar regions of interest, namely, the middle frontal gyrus (F2) and the AG.

The process of literature curation involves data entry into the matrix from publications reporting specific connections. By virtue of having been peer reviewed and published, an initial “curation” step has been performed by the scientific community itself. A sample schema and database user interface for entering all fields related to a connection (as described in Matrix and Proposed Database Design above) is shown in Figure 1.

## Results

We present both a pre-DTI era structural connectional matrix (online suppl. material, available at www.karger.com/doi/10.1159/000530358) and a proposed DTI era framework for connectional information using two exemplar cortical regions. For the pre-DTI era, connectional data related to 49 cortical, 7 thalamic, and 10 striatal regions, as described by Makris et al. [4], as well as connections of the pons, hippocampus, and amygdala have been collated. A database and website design for this information has been proposed (Fig. 1a, b). This website would allow identification of the connections of specific regions as well as the option to search for regions with specific connectional types.

Structural connections are characterized in terms of the anatomical pathway name. The sets of regions connected by the SLF, inferior longitudinal fascicle, cingulum bundle, occipito-frontal fascicle, extreme capsule, uncinate fascicle, internal capsule, corticopontine, corticostriatal, and amygdalofugal pathways are provided. Callosal connections generally are assumed to be present between each cerebral cortical region and its homotopical contralateral region. Although heterotopical callosal connections have been identified in the human brain (e.g., [10, 30]), for simplicity these are not considered herein.

**Fig. 3.**
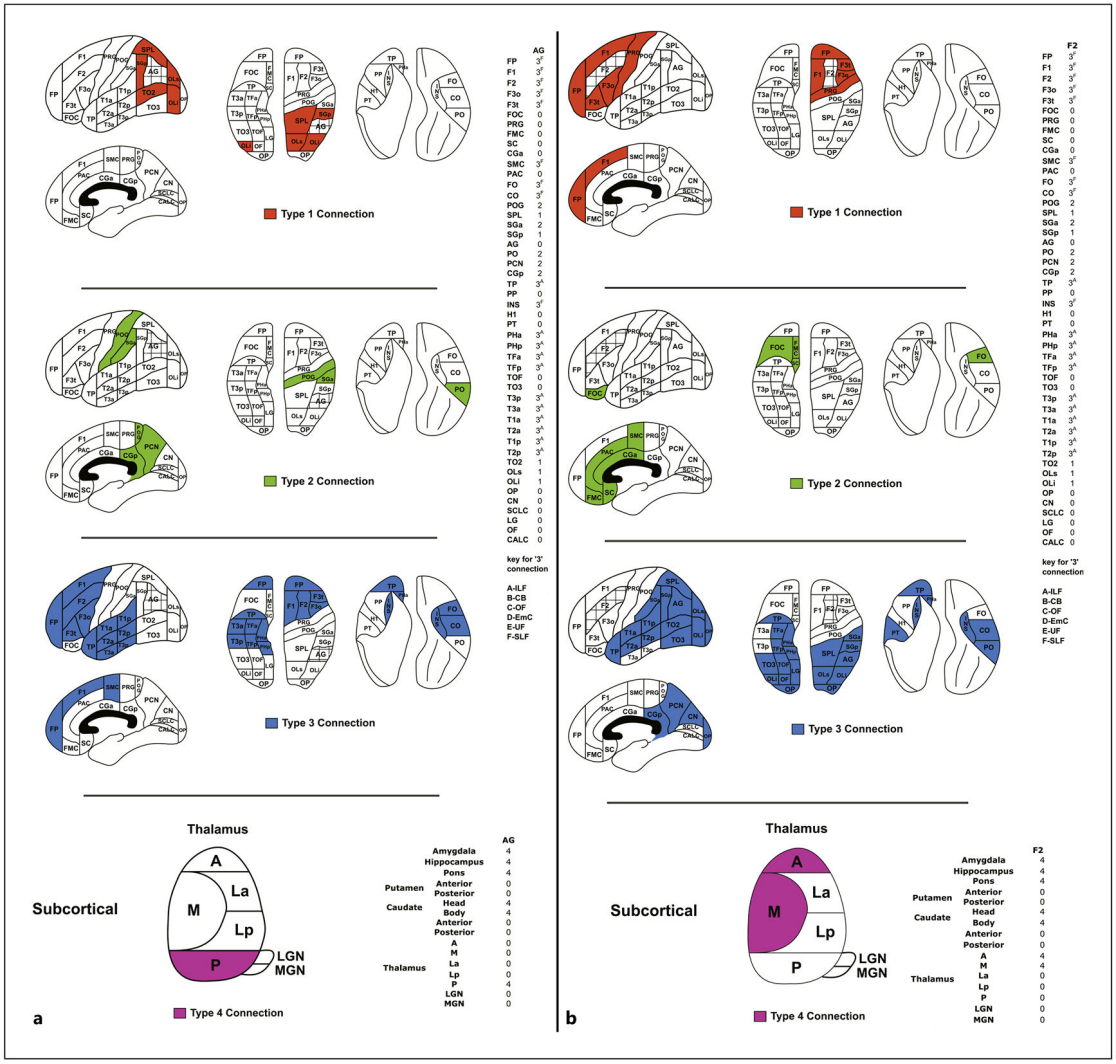
**a** Example of an anatomic connectivity map for the angular gyrus (AG). Colored regions show the type of connection to AG. PUs in red (first panel) are connected via a distance type 1 connection; green (second panel) via a distance type 2 connection; blue (third panel) via a distance type 3 connection. On the right, the cortical connectivity is shown in tabular form. Additionally, each connection via type 3 is characterized by its specific association fiber bundle. Type 4 connections (bottom panel) are specified to subcortical regions in a matrix (right) and in an overview of the thalamus (left). For cerebral cortical parcellation unit abbreviations, see Table 1. ILF, inferior longitudinal fascicle; CB, cingulum bundle; OF, occipito-frontal fascicle; EmC, extreme capsule; UF, uncinate fascicle; SLF, superior longitudinal fascicle; A, anterior thalamic parcellation unit; M, medial thalamic parcellation unit; La, lateral anterior thalamic parcellation unit; Lp, lateral posterior thalamic parcellation unit; P, posterior thalamic parcellation unit; LGN, lateral geniculate nucleus thalamic parcellation unit; MGN, medial geniculate nucleus thalamic parcellation unit. **b** Example of an anatomic connectivity map for the middle frontal gyrus (F2). Colored regions show the type of connection to F2. PUs in red (first panel) are connected via a distance type 1 connection; green (second panel) via a distance type 2 connection; blue (third panel) via a distance type 3 connection. On the right, the cortical connectivity is shown in tabular form. Additionally, each connection via type 3 is characterized by its specific association fiber bundle. Type 4 connections (bottom panel) are specified to subcortical regions in a matrix (right) and in an overview of the thalamus (left). For abbreviations, see Table 1 and above under **a**.

**Fig. 4.**
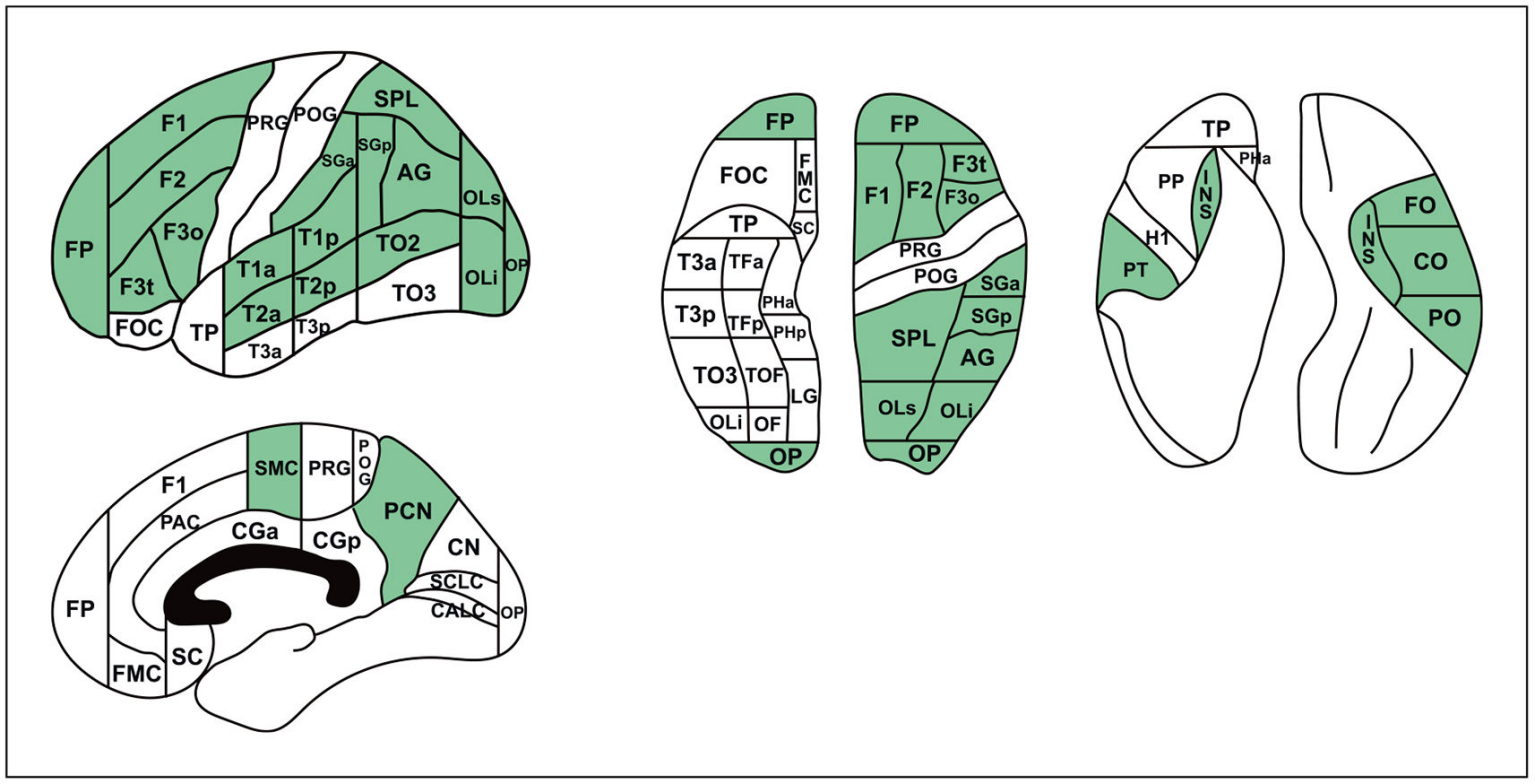
Colored regions show the PUs that are connected by a type 3 connection via the superior longitudinal fascicle (SLF) fiber bundle ([4, 10]; pre-DTI era). For abbreviations, see Table 1.

The structural connectivity map of a given cortical region of interest (ROI) is delineated based on its connectional typology. To illustrate this point, Figure 3 depicts an example of the canonical representation of two cortical regions, the AG (Fig. 3a) and the middle frontal gyrus (F2; Fig. 3b). These representations are shown pictorially, in terms of a generalized view of the cortical topography, and in “column matrix” form, indicating the presence (or absence) and type of connection between the structure of interest and other anatomic regions. For simplicity of visualization, coding of connections is as follows: type 1 is short-range U-fibers (typically juxtagyral) and intragyral connections; type 2 is medium-range intralobar connections; type 3 is long-range associational connections, and type 4 is projection (subcortical) connections. Figure 4 illustrates the information in the matrix from the perspective of a particular fiber system, namely, the SLF. The idealized pathway is coded relative to the cortical regions it interconnects, and reflects information from the pre-DTI era.

**Fig. 5.**
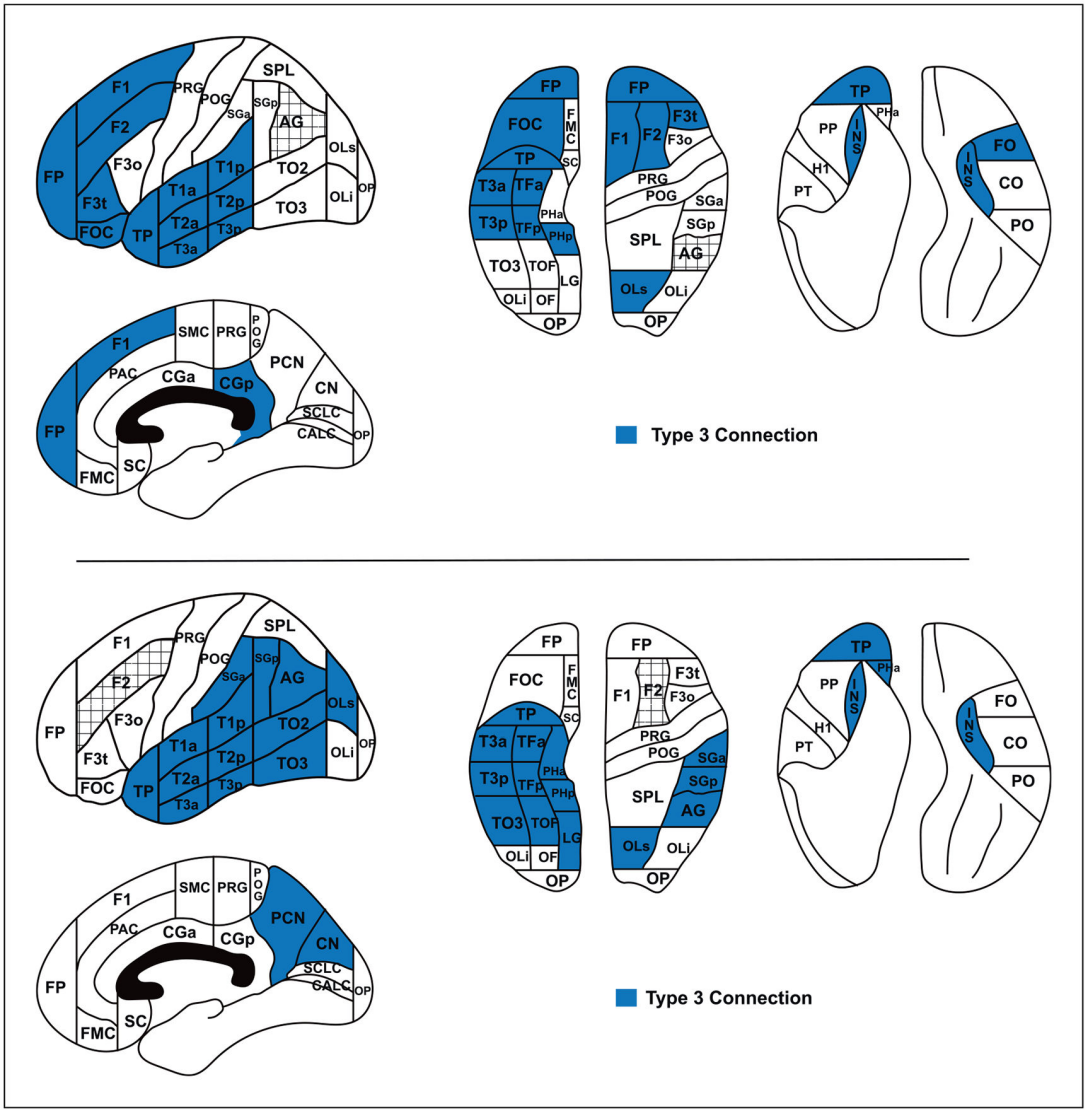
DTI era human structural connectivity maps for angular gyrus (AG, upper), and middle frontal gyrus (F2, lower) for type 3 connections. For abbreviations, see Table 1.

The results of the DTI era are illustrated in Figure 5 in two representative exemplar results for F2 and AG. A comparison of these data with the pre-DTI era data (Fig. 3) shows similarities as well as differences. For example, based on the classic Dejerine pre-DTI era connectivity matrix, the AG would connect with a greater number of cortical regions, including F3o, CO, SMC, and PHa (see Table 1). Furthermore, the middle frontal gyrus (F2) is connected with more regions in the pre-DTI era, including SPL, OLi, PT, CO, PO, and CGp (see Table 1). This result is understandable given that the DTI era approach is based on extrapolations from validated nonhuman primate structural connectivity information, which provides a more conservative estimate of the extent of type 3 connections [2, 45, 95, 96].

## Discussion

In the present study, we provide a macroscale level of inferred human structural cerebral connectivity for the pre-DTI era in the form of a connectivity matrix. This matrix reflects the knowledge base of human structural neuroanatomy derived from traditional histology and microdissection studies carried out largely prior to 2000. This matrix is expressed in terms of origins, terminations and topographical information about fiber tracts, and contextualized with respect to cortical PUs for applicability to MRI-based studies. We also present a framework for a DTI era human structural connectivity matrix. The DTI era matrix is different from the pre-DTI era matrix in that the former is constrained by validated nonhuman primate structural connectivity information with respect to the origins and terminations of the individual fiber tracts as well as by DTI tractography-based observations in the human and nonhuman primate brain. We illustrate differences between the two matrices using two exemplar cortical areas, namely, the AG and middle frontal gyrus (F2). In both the pre-DTI era and DTI era matrices, we use a neuroanatomical typology to characterize different types of connections in the human brain, which provides a critical dimension for organizing the matrices and the prospective database. This type of information provides a framework for the creation of more complete DTI era matrices going forward. Both pre-DTI and DTI era structural connectivity data are important sources of information that can be utilized in clinical neuroscience.

### Overview of the Matrix and Anatomic Brain Connectivity

The proposed structural connectivity matrix opens new windows of opportunity in the field of MRI brain volumetrics [5] and neurology. To date, stroke neurology has focused mainly on investigations of vascular territories related to stroke. In the words of the late Dr. Verne Caviness:

“Herein we envision that neurology could engage with four ontologies, that is, four realities that we don’t know exactly to what extent they are related and may be translatable to each other. One is the anatomical reality, which we can directly observe and characterize by T1-MRI-based analysis of lesions. Another is the connectionist reality that one might characterize by DTI. The third is the rather abstract, functionalistic one, which can be represented by fMRI. For instance, if the arcuate fascicle is damaged, there may emerge certain patterns from DTI that would conform with fMRI and others that would not. However, there are these two realities, i.e., the connectional and the functional, that should have some correlation. Finally, the fourth ontology is the behavioral, i.e., the clinical one. Now, if we could map these four realities onto each other, then there would be several avenues opened up. This matrix of cerebral connections creates the opportunity to carry out this endeavor to its extreme, that is, to map these four realities to each other. Technically, this can be done by applying “filters” onto the connectional matrices. These filters would be based on functional and behavioral bases, namely there will be a functional and a behavioral filter. For example, in Alzheimer’s disease we could take the tested cognitive reality and try to predict what happened to the anatomical connectivity of the brain. Then we could extend this inquiry to the functional connectivity of the brain and then question how the systems organization falls apart in degenerative disease and how this may bear very little relationship to the way it is damaged by a focal lesion. And this is an interesting hypothesis that could be tested.”

From an anatomical perspective, the white matter in the brain contains axons subserving cortico-cortical, commissural, cortico-subcortical, and cerebellar connections via associational, commissural, and projection fiber systems. Morphologically, we can distinguish three different components in a fiber bundle, namely (a) a compact portion or “stem,” where the axons course together to form a distinct fascicle; (b) a zone of “spray,” where the axons fan out; and (c) the distal or extreme, peripheral “origins” and “terminations” where the axons originate or end in gray matter (“extreme periphery”) (e.g., [4, 14]). Current spatial resolution capabilities of diffusion imaging techniques allow the delineation of fiber pathway stems reliably in humans (e.g., [14, 49, 155]). Ultimately, we need a system for delineating the different connectional characteristics of each fiber and pathway in its entirety, i.e., origin and termination as well as the stem corresponding to its trajectory. The issue of a complete description of the origin and termination of fiber pathways has been addressed effectively in experimental animals but not in the human brain, as discussed in detail by Rushmore et al. [2].

### Anatomic Connectivity Matrices and Maps

Different approaches have been used in an attempt to elucidate connections in the cerebrum in terms of their topographic arrangement (e.g., [14]) as well as in the form of matrices (e.g., [19]). Based on classic but limited postmortem anatomic studies in the human, our group has formulated an MRI-based topographic system of human structural cerebral connectivity and a comprehensive description of maps of human cerebral connections, which have been integrated in the context of a methodology for topographic characterization and quantification of the human forebrain white matter [4, 8, 14]. This pre-DTI era formulation was white matter-centric, that is, it is essentially a connectivity map of individual fiber tracts as shown by Makris et al. [4]. Although it provided a comprehensive volumetric, connectional, and topographic analysis of the white matter fiber tracts, it did not provide a systematic list of the connections for each cortical PU. In principle, this list of connections can be derived from the anatomical connectivity maps provided by Makris et al. [4]. A list of the connections of each gyral-based cerebral cortical PU in the human brain, as ascertained from pre-DTI era published literature, has been compiled in matrix form. We plan to update and modify this information based on more recent DTI era connectivity findings from both humans and nonhuman primates within a single database that can be accessed and queried via the internet (e.g., [156]). The resulting DTI era connectivity matrix will be designed to reflect, through a mechanism of continual updates, the body of data on experimental animal and human structural brain connectivity. Related efforts have cataloged the corpus of data on structural connectivity in the mouse [157] and the macaque [18-20, 158]. These are highly important resources for comprehensive cataloging of the wealth of information available in experimental animals. However, no comparable resource has been established for the human brain due to limitations on the validity of structural connectional information in the human brain, as elaborated in detail by Rushmore et al. [2].

### Assessment of Fiber Tracts Using DTI in Human

The advent of DTI tractography has enabled the structural study of fiber tracts in the living human brain and has opened a new window on structural-functional and anatomic-clinical relationships (e.g., [4, 13-15, 159-162]). DTI analysis enables the characterization of a white matter fiber pathway in terms of its orientation, location, and size. The field of DTI-based brain analysis has expanded rapidly with impressive results. However, there remain basic conceptual obstacles to be overcome, as was addressed in detail by our group more than two decades ago [4, 14, 97, 155] and again more recently [2]. Specifically, current diffusion imaging analysis techniques are able to identify and characterize reliably only the stems of the major fiber tracts (e.g., [14, 160-162]). The problem of elucidating the precise origins and terminations (what we term the extreme peripheries) of fiber tracts remains to be solved pending resolution of issues of acquisition, i.e., high angular resolution (e.g., [163]), spatial resolution (e.g., [164, 165]), tract modeling (e.g., [166]), and validation (e.g., [2, 14, 167]).

### The Macroscale Description of Brain Architecture

In both monkey and human brains, the macroscale level of description is currently approachable noninvasively and in vivo. This is an important level of description as numerous techniques (e.g., anatomic segmentation and parcellation, DTI, fMRI) commonly allow observations at this level, facilitating the integration of structural, functional, and behavioral correlates. A comprehensive matrix of the macroscale level architecture is key to the integration of these different classes of information.

### Utility of the Human Anatomical Connectivity Matrix

The matrix formulation of human anatomical cortical connectivity proposed herein may serve several purposes. These include predicting the effects of brain lesions on remote brain regions connected with the lesioned area, validating DTI and high angular resolution diffusion imaging (HARDI) data [163], as well as aiding a multitude of functional and metabolic brain investigations where anatomic connections are relevant (see, e.g., [155]). To illustrate these capabilities, we elaborate on a specific application as follows.

### Remote Effects of Brain Lesions – Lesion Analysis and Predictions

Knowing how a lesion or a brain structure, represented in neuroimaging as a ROI, interacts with the rest of the brain is an important yet often unanswered question in clinical neuroscience. Each ROI is connected with the rest of the brain in different ways in terms of classes of connections and numbers of fibers. These connections are organized so that the “small-world” properties (e.g., [168]) of the brain can be effected efficiently. This qualitative schema of structural anatomical connectivity needs to be translated to a quantitative expression of the relative influence or weightings of these different fibers (e.g., [169, 170]).

**Fig. 6.**
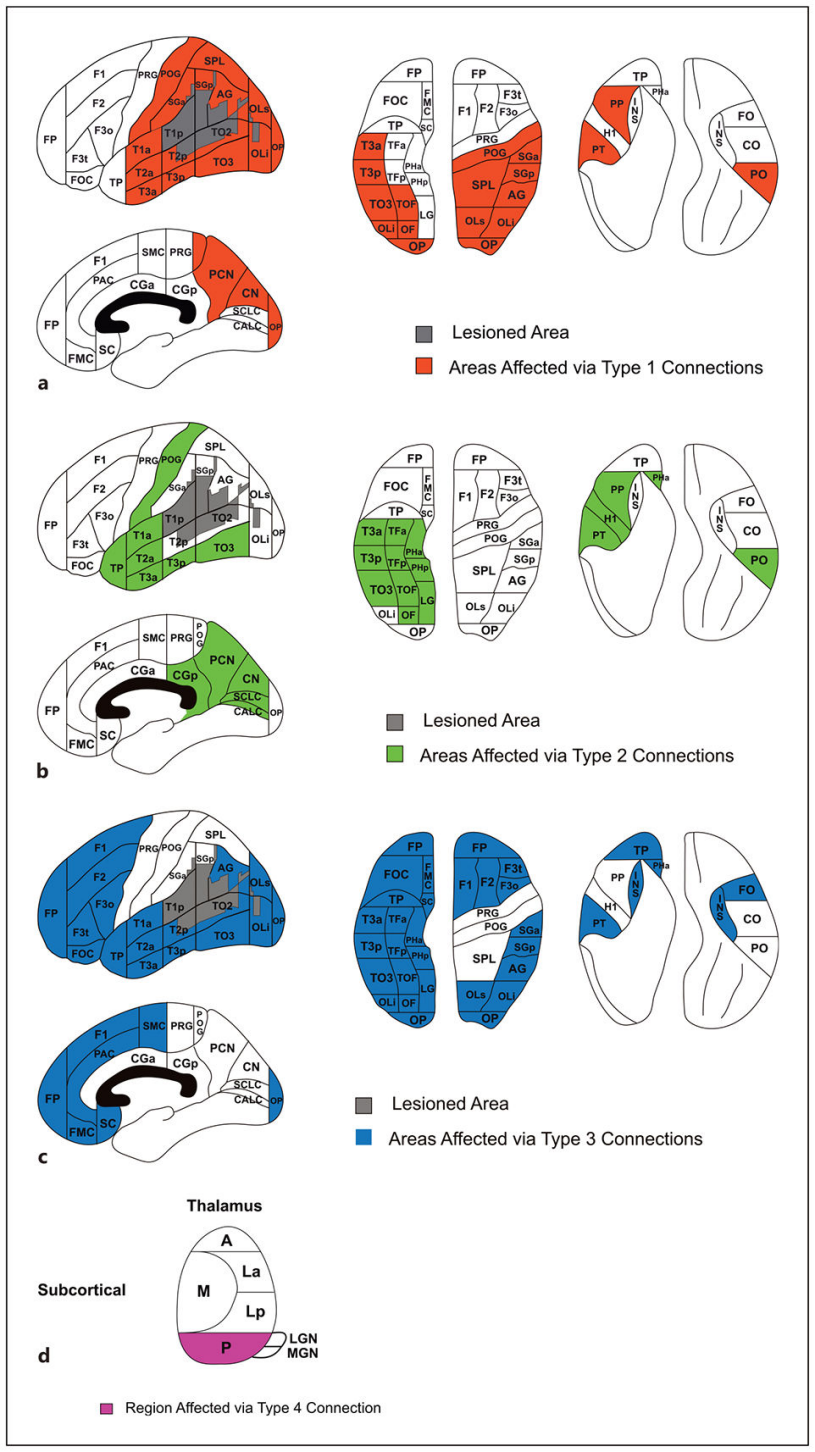
Colored regions show effect of stroke through type 1 (**a**), type 2 (**b**), type 3 (**c**), and type 4 (thalamic only; **d**) connections to the lesioned area (in gray). Given that the lesion occupies multiple PUs, we present here for simplicity a cumulative representation of the connectivity pattern for each fiber type. Ideally, each parcellation unit involved in a specific lesion would have its own connectivity map, as shown in Fig. 3. For a detailed description of distance type, refer to the Connection Class and Distance Type section of the Methods; for abbreviations, see Table 1.

As a practical application that utilizes the connectional matrix proposed herein, the set of remote regions putatively disconnected as a result of a stroke in a single case study is shown in Figure 6. This figure depicts areas affected via disconnection from a lesioned cortical region based on pre-DTI era knowledge of intrahemispheric association connections. We differentiate between the impact of the lesion conveyed through short-range (type 1), medium-range (type 2), and long-range association (type 3) fibers. A putative map of the effects of such altered anatomic connectivity (e.g., [9, 171-174]) in remote cortical ROIs is shown in Figure 6 [14, 155].

In this sample application, we integrate connectional information with the anatomical lesion caused by a cerebral infarction. The stroke causes direct destruction of specific regions of the cerebral cortex. The regions directly affected by the stroke are interconnected with other cortical and subcortical (type 4; only thalamic shown) regions via numerous fiber tracts. Note that this sample application based on pre-DTI era structural connectivity information is not definitive. Rather, it represents a starting point to illustrate proof of principle and utility for the empirical study of how best to construct a framework, based on structural connectivity findings, to study the remote effects of disconnection.

### Sources of Connectional Information and Curation of Data

The structural connectional information captured in the current version of our pre-DTI era matrix comes from human studies (e.g., [9, 10]) based on fiber degeneration methods (e.g., [23-30]); fiber microdissection preparations (e.g., [11, 22]); and a variety of atlases in humans (e.g., [10, 57, 106, 175]). The DTI era structural connectivity matrix will include connectional information from pre-DTI era as well as more current DTI findings informed by, and where possible validated by, nonhuman primate experimental findings (see, e.g., [2, 4, 45, 50, 97]).

### The Collation of Connectivity Data for the Macaque Database System

Based on literature mining, curation, and databasing, the Collation of Connectivity Data for the Macaque connectivity matrix has been generated for the nonhuman primate cerebrum [18-20, 158]. Collation of Connectivity Data for the Macaque collates and integrates published data on neuronal connectivity in the macaque from a variety of methods as well as information from different brain parcellation mapping schemas. It is a database for integrating primate brain connectivity based on principles of objectivity, reproducibility, transparency, flexibility, and applicability [18-20, 158]. It provides information on the topological organization of large-scale cortical networks in the monkey brain and the laminar origins and terminations of individual projections as well as structural cerebral cortical networks [176]. This database, which was derived from a large body of published literature, is very important for network analysis and modeling. Although such a database is feasible in macaque monkeys due to the detailed delineation of brain connections, including origins and terminations as well as stems, it is currently not possible in humans at this same level of detail (see, e.g., [2, 16]). Our present matrix for the human brain aims to generate a similar connectional database using a specific reference system of human cerebral anatomy [65, 93].

### Limitations and Future Directions

The elucidation of comparative structural brain connectivity in experimental animals and humans is of critical importance (e.g., [1]). Ideally, a structural connectivity matrix should include the origins and terminations of each pathway. Furthermore, the brain circuit diagram or BCD [2] should include a spatial representation of the fiber tracts that provides the exact topography of their trajectories in relation to origins and terminations. Moreover, an additional dimension of the BCD should be quantitative information on the fiber tracts, such as cross-sectional area, volume, and biophysical parameters (e.g., fractional anisotropy, FA; kurtosis), which would allow statistical analysis and modeling. Given the noninvasive nature of DTI and its in vivo applicability in human subjects, we expect DTI tractography [177, 178] to play a critical role in both the topographical and quantitative characterization of human brain structural connectivity (as illustrated in Fig. 2). We expect that the DTI era structural connectivity matrix will become more inclusive and complete as the methodology and findings on type 1 and type 2 connections in humans and nonhuman primates continue to advance.

We realize that any single formulation of typology such as that in the present paper represents only a starting point. We expect this formulation to be refined and advanced as our knowledge of anatomical structural connectivity grows over time and is integrated with functional and computational measures. Thus, modified or wholly new ontologies may be created to embody our evolving understanding of brain organization. Future studies, in both humans and experimental animals, should address more fully all of the critical dimensions of structural connectivity including origin and termination as well as trajectory. More complete, finer-grained anatomical frameworks and increasingly sophisticated methodologies will need to be developed with the goal of delineating human brain connectivity homologically and, ultimately, directly.

## Supplementary Material

Supplementary Table S2

Supplementary Table S1

## Figures and Tables

**Table 1.** Parcellation units

AG	Angular gyrus
CALC	Intracalcarine cortex
CGa	Cingulate gyrus, anterior division
CGp	Cingulate gyrus, posterior division
CN	Cuneal cortex
CO	Central opercular cortex
F1	Superior frontal gyrus
F2	Middle frontal gyrus
F3o	Inferior frontal gyrus, pars opercularis
F3t	Inferior frontal gyrus, pars triangularis
FMC	Frontal medial cortex
FO	Frontal opercular cortex
FOC	Frontal orbital cortex
FP	Frontal pole
H1	Heschl’s gyrus
INS	Insula cortex
LG	Lingual gyrus
OF	Occipital fusiform gyrus
OLi	Lateral occipital cortex, inferior
OLs	Lateral occipital cortex, superior
OP	Occipital pole
PAC	Paracingulate gyrus
PCN	Precuneal cortex
PHa	Parahippocampal gyrus, anterior
PHp	Parahippocampal gyrus, posterior
PO	Parietal opercular cortex
POG	Postcentral gyrus
PP	Planum polare
PRG	Precentral gyrus
PT	Planum temporale
SC	Subcallosal cortex
SCLC	Supracalcarine cortex
SGa	Supramarginal gyrus, anterior
SGp	Supramarginal gyrus, posterior
SMC	Supplementary motor Cortex
SPL	Superior parietal lobule
T1a	Superior temporal gyrus, anterior
T1p	Superior temporal gyrus, posterior
T2a	Middle temporal gyrus, anterior
T2p	Middle temporal gyrus, posterior
T3a	Inferior temporal gyrus, anterior
T3p	Inferior temporal gyrus, posterior
TFa	Temporal frontal cortex, anterior
TFp	Temporal frontal cortex, posterior
TO2	Middle temporal gyrus, temporo-occipital
TO3	Inferior temporal gyrus, temporo-occipital
TOF	Temporal occipital fusiform cortex
TP	Temporal pole

**Table 2.** Typology of structural cerebral cortical connections

Distance type	Intra- or interlobar	Adjacent areas connected?	Areas within the same lobe connected?	Regions across lobes connected?
Type 1 connections				
Short-range	Intralobar	Y	Y	N
Short-range	Interlobar (juxtalobar)	Y	N	Y
Type 2 connections				
Medium-range	Intralobar	N	Y	N
Type 3 connections				
Long-range	Interlobar	N	N	Y

## Data Availability

All data generated or analyzed during this study are included in this article and its online supplementary material files. Further inquiries can be directed to the corresponding author.
